# Phenolic Compounds from the Roots of *Rhodiola crenulata* and Their Antioxidant and Inducing IFN-γ Production Activities

**DOI:** 10.3390/molecules200813725

**Published:** 2015-07-28

**Authors:** Jiang-Tao Zhou, Chen-Yang Li, Chun-Hua Wang, Yue-Fei Wang, Xiao-Dong Wang, Hong-Tao Wang, Yan Zhu, Miao-Miao Jiang, Xiu-Mei Gao

**Affiliations:** 1 Tianjin Key Laboratory of Modern Chinese Medicine, Tianjin University of Traditional Chinese Medicine, Tianjin 300193, China; E-Mails: zjt19881206@126.com (J.-T.Z.); pharmwch@126.com (C.-H.W.); 13516185421@139.com (Y.-F.W.); yanzhu.harvard@gmail.com (Y.Z.); gaoxiumei@tjutcm.edu.cn (X.-M.G.); 2 Research and Development Center of Traditional Chinese Medicine, Tianjin International Joint Academy of Biotechnology & Medicine, Tianjin 300457, China; 3 Department of Pharmacy, School of Medicine, Shenzhen University, Shenzhen 518060, China; E-Mails: lichenyangchina@163.com (C.-Y.L.); wangxiaodong@szu.edu.cn (X.-D.W.); 4 Shijiazhuang Yiling Pharmaceutical Co., Ltd, Shijiazhuang 050035, China; E-Mail: wht72007@126.com

**Keywords:** *Rhodiola crenulata*, phenol, antioxidant activity, IFN-γ production, immunomodulatory effect

## Abstract

In the present study, two new phenolic compounds **1** and **11**, a pair of lignan isomers **12** and **13** with their absolute configurations established for the first time, were isolated from the ethanol extract of the roots of *Rhodiola crenulata*, together with 13 known phenolic compounds, and their structures were elucidated via NMR, HRESIMS, UV, IR and CD analyses. All the isolated compounds were evaluated for their *in vitro* antioxidant activities using the 2,2-diphenyl-1-picryhydrazyl (DPPH) and 2,2′-azino-bis (3-ethylbenzothiazoline-6-sulfonic acid) (ABTS) radical scavenging assays. Ten of them exhibited significant antioxidant activities compared to ascorbic acid. Furthermore, the inducibilities of the isolated compounds to IFN-γ production were also assessed. Compounds **1**, **8**, **9**, **12**, **13**, **14** and **15** could moderately stimulate IFN-γ expression.

## 1. Introduction

*Rhodiola crenulata* (HK. f. *et*.Thoms) H. Ohba (Dhua Hong-Jing-Tian in Chinese) is an important species among the *Rhodiola* genus, mainly found distributed in western regions of China including the provinces of Yunnan, Sichuan and Tibet [1]. As a functional food, *R. crenulata* is used for prevention against high-altitude illness and regarded by the tourists going to plateau as a traditional phyto-adaptogen to environmental challenges [2]. Moreover, the extracts of *R. crenulata* have been made into pharmaceutical preparations and cosmetics with varied bioactivities [3,4]. Due to its extensive application in food, medicine and cosmetics, the chemical constituents and pharmacological activities of *R.* *crenulata* have been widely investigated. The main chemical constituents of *R. crenulata* are phenolic compounds, such as flavonoids, phenylpropanoids, phenolic acids and so on [5,6,7,8,9,10]. Modern pharmacological investigations have revealed that *Rhodiola* preparations exhibit antioxidant, immunomodulatory, anti-aging, anti-fatigue [11,12], neuroprotective [13], anti-inflammatory [14], antidepressive, anxiolytic, nootropic, life-span increasing and central nervous system (CNS) stimulating activities [15]. Due to the diverse biological activities of *R. crenulata*, the chemical constituents of *R. crenulata* and the bioactivities of the isolates were investigated by our research group.

## 2. Results and Discussion

### 2.1. Identification of Compounds **1**–**17**

**Figure 1:**
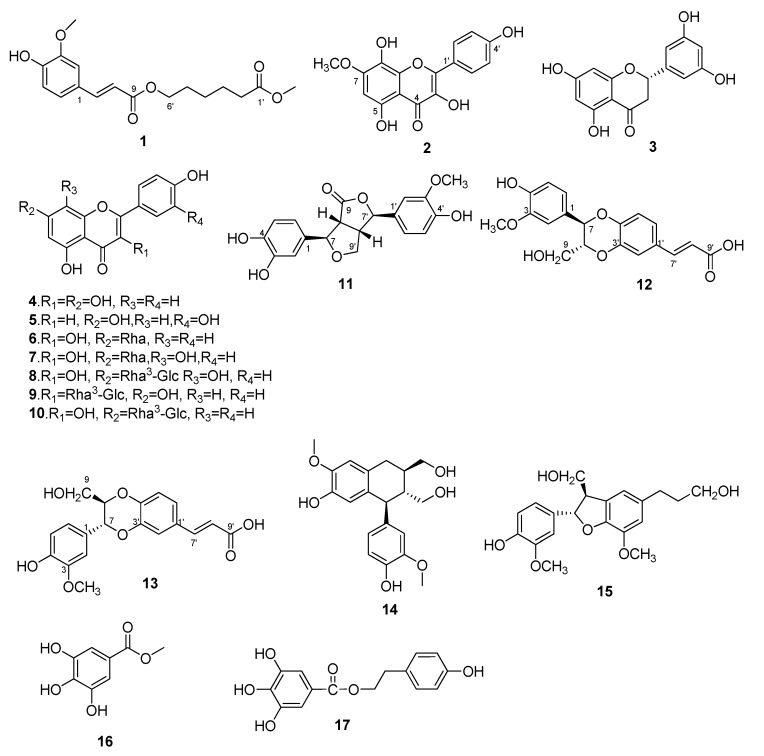
Chemical structures of compounds **1**–**17**.

**Figure 2:**
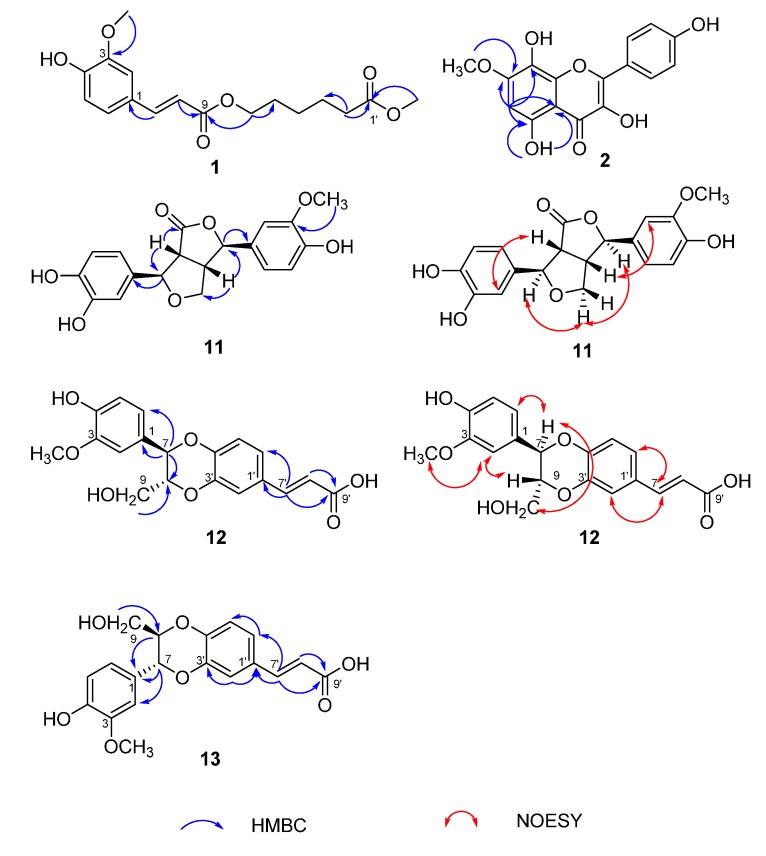
Key HMBC or NOESY correlations of compounds **1**, **2** and **11**–**13**.

Compound **1** was isolated as a light yellow solid. Its molecular formula was determined as C_17_H_22_O_6_ by HRESIMS [M − H]^−^ at *m*/*z* 321.1338, (calcd, for C_17_H_21_O_6_, 321.1338). The IR spectrum displayed OH (3411 cm^−1^), C=O (1708 and 1734 cm^−1^), and C=C (1633 cm^−1^) functions. The ^1^H-NMR spectrum revealed the existence of five methylenes at δ_H_ 4.12 (2H, t, *J* = 6.6 Hz), 2.33 (2H, t, *J* = 7.4 Hz), 1.64 (2H, m), 1.58 (2H, m) and 1.37 (2H, m), an ABX aromatic protons at δ_H_ 7.33 (1H, d, *J* = 2.0 Hz), 7.12 (1H, dd, *J* = 8.2, 2.0 Hz) and 6.80 (1H, d, *J* = 8.2 Hz), and two methoxyls at δ_H_ 3.83 and 3.59. The signals at δ_H_ 7.55 (1H, d, *J* = 16.0 Hz) and 6.47 (1H, d, *J* = 16.0 Hz) suggested *trans* double bond protons. The ^13^C-NMR spectrum revealed the presence of 17 C-atoms, which were identified with DEPT-135 spectrum as five methylenes (δ_C_ 63.5, 33.1, 27.9, 25.0 and 24.1), two methoxyls (δ_C_ 55.7 and 51.2), two carbonyl groups (δ_C_ 173.3 and 166.7), as well as a pair of olefinic carbons with *trans* double bond features (δ_C_ 145.0 and 114.4). The chemical shifts from δ_C_ 111.2 to 149.5 were in the aromatic region, which indicated a benzene ring and a double bond existed. From the above NMR data (Table 1), a 3,4-disubstituted cinnamoyl group linked with a fatty alcohol was deduced. Thus, the chemical structure illustrated in Figure 1 were established on the basis of these data. This was further confirmed by the key HMBC correlations (Figure 2) from δ_H_ 3.59 (OCH_3_) to 173.3 (C-1′), from δ_H_ 4.12 (H-6′) to δ_C_ 166.7 (C-9) and 27.9 (C-5). So, compound **1** was determined as methyl 6-*O*-(3-methoxy-4-hydroxy-cinnamoyl)-caproate and named rhodiolate.

**Table 1:** ^1^H-NMR and ^13^C-NMR data of compounds **1**, **2** and **11**–**13** (400 MHz and 100 MHz, DMSO-*d*_6_, δ in ppm, *J* in Hz).

No.	1		2		11		12		13	
	δ_H_	δ_C_	δ_H_	δ_C_	δ_H_	δ_C_	δ_H_	δ_C_	δ_H_	δ_C_
1		125.5				131.8		127.7		127.6
2	7.33 (d, 2.0)	111.2		147.6	6.79 (d, 1.2)	114.0	7.01 (d, 1.8)	112.2	7.07 (s)	112.2
3		149.5		135.9		147.4		148.1		148.3
4		148.0		176.9		145.4		147.6		147.9
5	6.80 (d, 8.2)	115.5		152.7	6.74 ^a^	115.8	6.80 (d, 8.0)	115.8	6.84 ^a^	115.5
6	7.12 (dd, 8.2, 2.0)	123.1	6.56 (s)	95.4	6.64 (dd, 8.2, 1.8)	117.3	6.85 (dd, 8.0, 1.8)	121.0	6.85 ^a^	120.0
7	7.55 (d, 16.0)	145.0		154.0	5.07 (d, 3.6)	83.3	4.95 (d, 7.8)	76.6	4.90 (d, 7.8)	76.5
8	6.47 (d, 16.0)	114.4		126.4	3.70 (dd, 9.2, 3.8)	53.0	4.20 (m)	78.4	4.16 (m)	78.5
9		166.7		144.2		177.7	3.55 (d, 11.6) 3.35 ^a^	60.6	3.53 (d, 10.2) 3.35 (dd, 12.0, 4.2)	60.6
10				103.8						
1′		173.3		122.3		131.0		128.2		130.5
2′	2.33 (t, 7.4)	33.1	8.14 (d, 8.8)	130.2	6.99 (d, 1.2)	111.1	7.30 (d, 2.0)	116.9	7.05 (d, 2.0)	115.9
3′	1.58 (m)	24.1	6.94 (d, 8.8)	115.9		148.2		144.3		144.4
4′	1.37 (m)	25.0		159.8		145.7		146.1		143.8
5′	1.64 (m)	27.9	6.94 (d, 8.8)	115.9	6.81 ^a^	115.9	6.94 (d, 8.0)	117.6	6.87 (d, 8.0)	117.3
6′	4.12 (t, 6.6)	63.5	8.14 (d, 8.8)	130.2	6.85 (dd, 8.2, 1.8)	119.5	7.21 (dd, 8.0, 2.0)	122.1	7.00 (dd, 8.0, 1.8)	121.6
7′					5.39 (d, 3.6)	85.4	7.52 (d, 16.0)	143.9	7.12 (d, 15.8)	136.6
8′					3.28 (m)	49.3	6.38 (d, 16.0)	117.7	6.29 (d, 15.8)	127.6
9′					4.14 (dd, 9.2, 7.2) 3.95 (dd, 9.2, 4.0)	72.5		168.3		171.2
3-OCH_3_	3.83 (s)	55.7					3.78 (s)	56.1	3.76 (s)	56.2
5-OCH_3_			3.91 (s)	56.8						
1′-OCH_3_	3.59 (s)	51.2								
3′-OCH_3_					3.79 (s)	56.2				

^a^ Overlapped signals are reported without designating multiplicity.

Compound **2** showed an [M + H]^+^ ion peak at *m*/*z* 317.0667 (calcd. for C_16_H_13_O_7_, 317.0661) in the HRESIMS spectrum, consistent with the molecular formula of C_16_H_12_O_7_. The IR spectrum displayed OH (3418 and 3309 cm^−1^), C=C (1662 cm^−1^) and MeO (1252 cm^−1^) bands. The ^1^H-NMR spectrum revealed the existence of four active hydrogen protons at δ_H_ 12.01, 10.11, 9.40 and 8.75, and a pair of double peaks (δ_H_ 8.14 (2H, d, *J* = 8.8 Hz) and 6.94 (2H, d, *J* = 8.8 Hz)) ascribed to a 1,4-disubstituted benzene ring, as well as one single peak at δ_H_ 6.56. The ^13^C-NMR spectrum revealed the presence of 16 C-atoms, including 15 aromatic carbons at δ_C_ 176.9, 159.8, 154.0, 152.7, 147.6, 144.2, 135.9, 130.2, 130.2, 126.4, 122.3, 115.9, 115.9, 103.8 and 95.4, and a MeO at δ_C_ 56.8. Comparison the NMR data (Table 1) of compound **2** with those of herbacetin [16] revealed that the NMR data of compound **2** were similar to those of herbacetin, except for an extra MeO group. Based on the above NMR data, a MeO group substituting the OH at C-7 was proposed. This was further supported by HMBC correlation (Figure 2) between δ_H_ 3.91 (OCH_3_) and δ_C_ 154.0 (C-7). When comparing its ^13^C-NMR data to the previously published data of herbacetin 7-methyl ether [17], wrong assignments of C-5 (δ_C_ 153.72), C-7 (δ_C_ 159.39) and C-9 (δ_C_ 152.40) were noted in those published results. Consequently, compound **2** was identified as herbacetin 7-methyl ether, and its ^13^C-NMR data were corrected (see Table 1).

The molecular formula of compound **11** was determined as C_19_H_18_O_7_ by its HRESIMS [M + Na]^+^ signal at *m*/*z* 381.0953 (calcd. for C_19_H_18_O_7_Na, 381.0950). The IR absorptions suggested the existence of OH (3402 cm^−1^), C=O (1755 cm^−1^) and C=C (1650 cm^−1^) functionalities. The chemical shifts from δ_H_ 6.99 to 6.64 were in the aromatic region in the ^1^H-NMR spectrum, which indicated the existence of two 1,3,4-trisubstituted benzene systems (δ_H_ 6.79 (1H, d, *J* = 1.2 Hz), 6.74 (1H, overlapped) and 6.64 (1H, dd, *J* = 8.2, 1.8 Hz), and 6.99 (1H, d, *J* = 1.2 Hz), 6.85 (1H, dd, *J* = 8.2, 1.8 Hz) and 6.81 (1H, overlapped)), furthermore, a pair of oxymethylene signals (δ_H_ 4.14 (1H, dd, *J* = 9.2, 7.2 Hz) and 3.95 (1H, dd, *J* = 9.2, 4.0 Hz)), two oxymethine signals (δ_H_ 5.39 (1H, d, *J* = 3.6 Hz) and 5.07 (1H, d, *J* = 3.6 Hz)) were also visible in the ^1^H-NMR spectrum. The ^13^C-NMR spectrum displayed 19 C-atom signals, including two benzene rings (δ_C_ 147.4, 145.4, 131.8, 117.3, 115.8 and 114.0) and (δ_C_ 148.2, 145.7, 131.0, 119.5, 115.9 and 111.0), a methylene (δ_C_ 72.5), four methines (δ_C_ 85.4, 83.3, 53.0 and 49.3) and an ester carbonyl carbon (δ_C_ 177.7). The NMR data of **11** (Table 1) were closely similar to those of 4-ketopinoresinol [18], the chemical structure with the loss of MeO at C-3 was deduced on the basis of these data. This was further confirmed by the key HMBC correlation (Figure 2) from δ_H_ 3.79 (OCH_3_) to δ_C_ 148.2 (C-3′). The relative configuration was established by NOESY correlations (Figure 2) between δ_H_ 3.70 (H-8)/6.79 (H-2), δ_H_ 5.07 (H-7)/3.95 (H-9′b)/5.39 (H-7′), δ_H_ 3.28 (H-8′)/6.99 (H-2′). However, compound **11** gave a positive specific rotation ${\left[\alpha \right]}_{\text{D}}^{25}$ +4.97 (*c* 0.2, MeOH), opposite that of (−)-3,4,3′,4′-tetrahydroxy-9,7′β-epoxylignano-7β,9′-lactone (${\left[\alpha \right]}_{\text{D}}^{25}$ −5.8 (*c* 0.1, MeOH)) [19]. Thus, compound **11** was identified as (7β,7′β,8α,8′α)-3′-methoxy-9-oxo-7,9′,7,9′-diepoxylignan-3,4,4′-triol.

**Figure 3:**
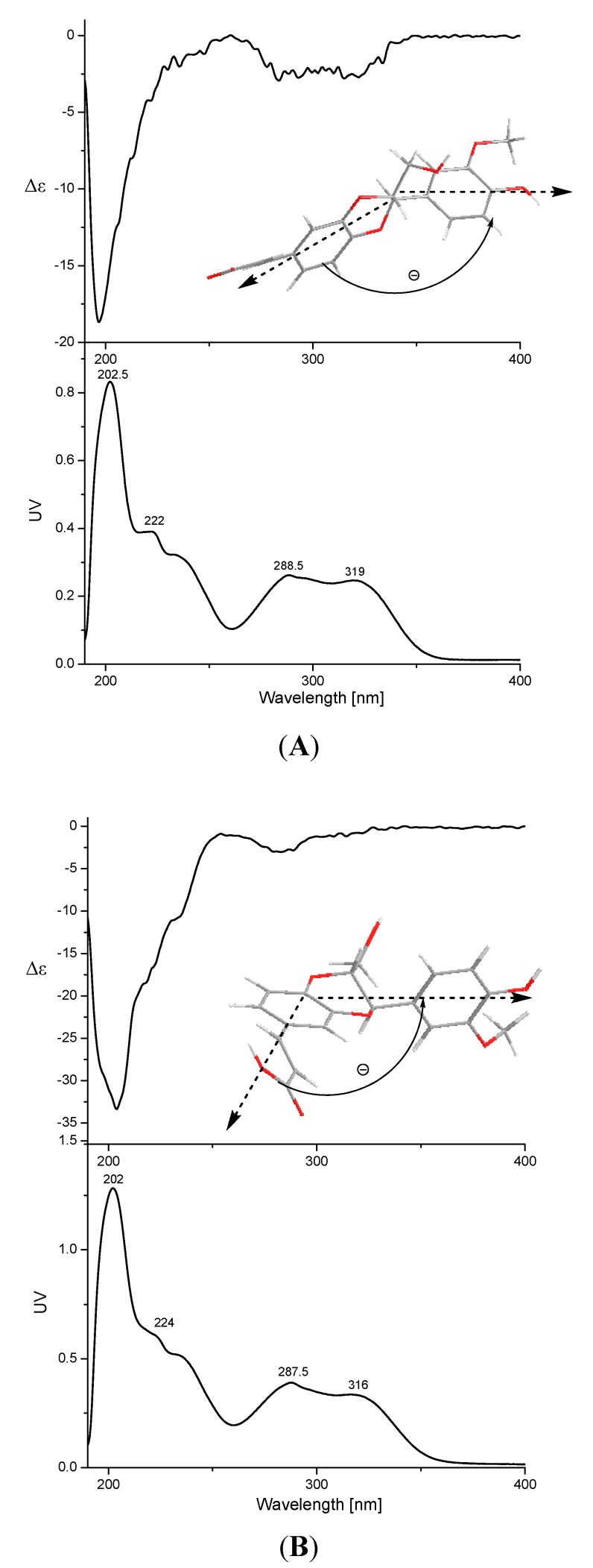
CD and UV spectra of compounds **12** (**A**) and **13** (**B**), where the arrow denotes the electronic transition dipole of the chromophores. (−) means the two chromophores should be oriented counterclockwise in space; (+) means the two chromophores should be oriented clockwise in space.

Compound **12** was isolated as a white amorphous powder, the HRESIMS of compound **12** showed an [M + Na]^+^ ion peak at *m*/*z* 381.0950 (calcd. for C_19_H_18_O_7_Na: 381.0950) for the molecular formula C_19_H_18_O_7_. The IR spectrum showed 3438 cm^−1^ (OH) and 1688 cm^−1^ (C=O) groups. The ^1^H-NMR spectrum of compound **12** displayed two pairs of ABX proton signals: δ_H_ 7.30 (1H, d, *J* = 2.0 Hz), 7.21 (1H, dd, *J* = 8.0, 2.0 Hz) and 6.94 (1H, d, *J* = 8.0 Hz), as well as δ_H_ 7.01 (1H, d, *J* = 1.8 Hz), 6.85 (1H, d, *J* = 8.0, 1.8 Hz) and 6.80 (1H, d, *J* = 8.0 Hz). A pair of proton signals (δ_H_ 7.52 (1H, d, *J* = 16.0 Hz) and 6.38 (1H, d, *J* = 16.0 Hz) of *trans*-double bonds were also seen in the ^1^H-NMR spectrum. Accordingly, the ^13^C-NMR and DEPT-135 spectra gave 19 C-atom signals. Except for a MeO group at δ_C_ 56.1, there remained 18 carbons, and a 3,4-disubstituted cinnamic acid (δ_C_ 168.3, 146.1, 144.3, 143.9, 128.2, 122.1, 117.7, 117.6 and 116.9) linked with a 7,8-disubstituted 3-methoxy-4-hydroxy-phenylpropanol (δ_C_ 148.1, 147.6, 127.7, 121.0, 115.8, 112.2, 78.4, 76.6 and 60.6) through a C-8-*O*-C-3′ and C-7-*O*-C-4′ linkage pattern was deduced. By comparison with the NMR data (Table 1) of compound **12** with those of arteminorin D [20], the data were seen to be the same as those of this compound. In the previous report, the absolute configuration of arteminorin D was not determined, so, the absolute configuration of compound **12** was elucidated by 2D NMR and CD exciton chirality methods in our work. The relative configuration (Figure 2) of compound **12** was identified by NOESY spectrum and the same as the literature reported (He *et al.*, 2009). The UV spectrum (Figure 3) of compound **12** showed an absorption at 222 nm attributable to the benzene moiety (π→π*). Corresponding to this UV absorption, the CD spectrum of compound **12** showed a negative Cotton effect at 222 nm due to the transition interaction between two different benzene moieties in the structure. The above information demonstrated a negative chirality for compound **12**, and the two aforementioned chromophores should be oriented counterclockwise in space (Figure 3). Thus, compound **12** was elucidated as (7*R*,8*R*)-3-methoxy-8′-carboxy-7′-en-3′,8-epoxy-7,4′-oxyneolignan-4,9-diol.

The HRESIMS of compound **13** showed an [M + Na]^+^ ion peak at *m*/*z* 381.0952 [M + Na]^+^ (calcd. for C_19_H_18_O_7_Na:381.0950) for the same molecular formula (C_19_H_18_O_7_) as that of compound **12**. Comparison the NMR data (Table 1) of compound **13** with those of compound **12** revealed that the NMR data of compound **13** were similar. A 3,4-disubstituted cinnamic acid linked with a 7,8-disubstituted 3-methoxy-4-hydroxy-phenylpropanol with different linkage patterns from that of compound **12** was deduced. Although no HMBC correlations from H-7 to C-3′ or H-8 to C-4′ were observed, the diagnostic chemical shifts of C-7 (δ_C_ 76.5), C-8 (δ_C_ 78.5), C-3′ (δ_C_ 144.4) and C-4′ (δ_C_ 143.8), combined with the established molecular formula, C_19_H_18_O_7_, suggested a linkage pattern of C-7-*O*-C-3′ and C-8-*O*-C-4′. Furthermore, a large coupling constant between H-7 and H-8 (*J* = 7.8 Hz) indicated a *trans* relationship of the two protons. This chemical structure (Figure 1) was reported in a Chinese patent [21] without the absolute configuration, so the absolute configuration of compound **13** was also elucidated using the CD exciton chirality method (Figure 3), and the absolute configuration of compound **13** was determined as (7*R*,8*R*). Thus, compound **13** was elucidated as (7*R*,8*R*)-3-methoxy-8′-carboxy-7′-en-3′,7-epoxy-8,4′-oxyneolignan-4,9-diol.

In addition, the other 12 known phenolic compounds were identified as 5,7,3′,5′-tetrahydroxy-dihydroflavone (**3**) [22], kaempferol (**4**) [23], luteolin (**5**) [24], kaempferol-7-*O*-α-l-rhamnoside (**6**) [23], rhodionin (**7**) [6], rhodiosin (**8**) [6], ternatumoside II (**9**) [25], crenuloside (**10**) [6], (+)-isolarisiresinol (**14**) [26], (+)-dihydrodehydrodiconiferyl alcohol (**15**) [27], methyl gallate (**16**) [28] and 2-(4-hydroxyphenyl) ethyl 3,4,5-trihydroxybenzoate (**17**) [29] by comparing their physical and spectral data with literature values.

### 2.2. Antioxidant Activities

The DPPH and ABTS radical-scavenging assays are effective methods used to evaluate the antioxidant activity of natural products. As shown in Table 2, new compound **1** exhibited no radical-scavenging activity, while compounds **2**–**10** showed potent radical-scavenging activities comparable to ascorbic acid, compounds **16** and **17** exhibited more intense radical-scavenging activities. In all, the radical-scavenging activity of compounds is also attributed to the hydroxyl groups substituted on the aromatic ring.

**Table 2:** The IC_50_ values in μM of antioxidant activities of **1**–**17** (*n* = 3).

Compounds	DPPH IC_50_ (μM) ^a^	ABTS IC_50_ (μM) ^a^
**1**	>500 ^b^	>500
**2**	114.3 ± 4.4	87.0 ± 7.9
**3**	73.8 ± 2.9	129.8 ± 12.3
**4**	91.6 ± 1.4	125.4 ± 9.8
**5**	94.3 ± 2.3	123.9 ± 10.4
**6**	83.0 ± 3.5	123.7 ± 12.8
**7**	104.7 ± 1.8	63.7 ± 8.5
**8**	96.5 ± 2.3	53.1 ± 4.8
**9**	260.5 ± 36.4	320.2 ± 22.6
**10**	64.1 ± 3.3	110.8 ± 10.7
**11**	>500	>500
**12**	>500	>500
**13**	>500	>500
**14**	227.1 ± 33.9	160.2 ± 8.8
**15**	>500	>500
**16**	52.8 ± 3.3	50.0 ± 4.9
**17**	79.5 ± 1.7	65.0 ± 3.9
**Ascorbic acid**	88.6 ± 1.9	89.8 ± 6.8

^a^ Data were represented as mean ± SD; ^b^ The IC_50_ value of sample is higher than 500 μM.

### 2.3. Compounds Treatment Stimulates IFN-*γ* Production Activities

**Figure 4:**
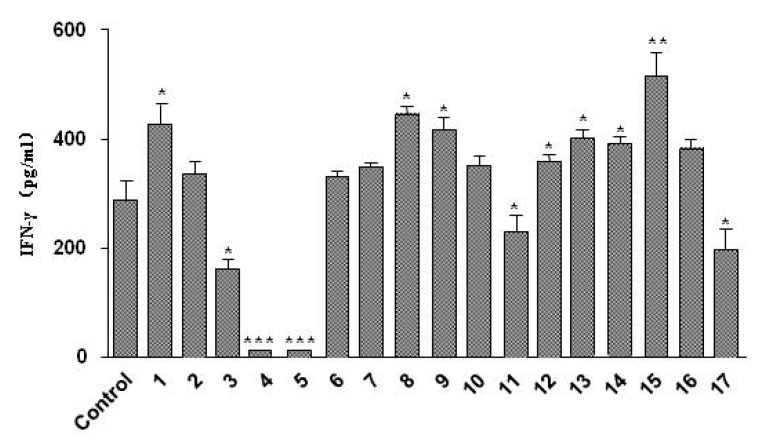
IFN-γ production by spleen lymphocyte cells treated with compounds **1**–**17**. Valus are means ± S.E.M., *n* = 3; * *p* < 0.05; ** *p* < 0.01; *** *p* < 0.005.

**Figure 5:**
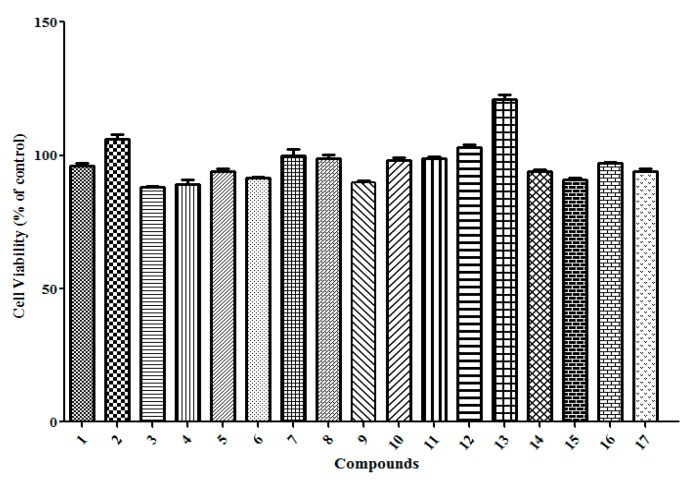
Cell viability of mouse spleen lymphocyte cells after 48 h treatment by 100 μM concentration of compounds **1**–**17** using CCK-8 assay (data points represent the mean ± S.D., *n* = 3).

As reported in the literature [30,31], nitric oxide production by activated macrophages *in vitro* and *in vivo* is dependent on IFN-γ, which is corresponding to the immunomodulatory effect [32]. To further evaluate the biological of these isolated phenolic compounds, the inducibility to IFN-γ release was measured in cell culture supernatants. As shown in Figure 4, compounds **1**, **8**, **9**, **12**, **13**, **14** and **15** could moderately stimulate IFN-γ expression. Finally, CCK-8 assays were used to determine if these isolates suppress the production of IFN-γ. As Figure 5 shows, there was no significant reduction in cell viability by these compounds (100 μM concentration). Therefore, we can conclude that the four lignans (compounds **12**, **13**, **14** and **15**) and the two flavonoid glycosides (compounds **8** and **9**) from *R. crenulata* have the ability of inducing IFN-γ production, moreover, the new compound **1** also possesses the inducing IFN-γ release activity. These compounds would be the active chemical constituents related to the immunomodulatory effect of *R. crenulata*.

## 3. Experimental Section

### 3.1. General Procedures

All reagents were of HPLC or analytical grade and were purchased from Tianjin Concord Chemical Company (Tianjin, China). Optical rotation values were measured by a P-1020 polarimeter (JASCO International Co., Ltd., Tokyo, Japan). IR spectra were measured on a Perkin Elmer spectrum 65 FT-IR spectrometer (PerkinElmer, Waltham, MA, USA). HRESIMS data were determined by an Agilent 6210 ESI/TOF mass spectrometer (Agilent Co., Santa Clara, CA, USA), the full scan mass spectra data were acquired in positive and negative ion modes. Acquisition parameters are as follows: capillary voltage was 3000 V for ESI (+) and 2600 V for ESI (−); cone voltage was 45 V; the ESI source temperature was 100 °C; the desolvation temperature was 350 °C; the nitrogen (N_2_) was used as desolvation gas at flow rates of 600 L/h for both ESI (+) and ESI (−); and the range of full scan was set at *m*/*z* 150–1000 Da.). NMR spectra were recorded on a Bruker-AVIII-400/600M spectrometer (Bruker Co., Geneva, Switzerland). CD spectra were recorded on a JASCO J-720W spectropolarimeter (JASCO). The ODs were recorded on a Flexstation 3 (Molecular Devices Co., Sunnyvale, CA, USA). Preparative HPLC: Agilent 1260 system equipped with a preparative Cosmosil C_18_ (5 μm, 20 mm × 250 mm) column (Agilent Co.). Column chromatography (CC): silica gel (SiO_2_; 200–300/400–500 mesh, Qingdao Marine Chemical Factory, Qingdao, China); Sephadex LH–20: (GE Healthcare UK Ltd, Buckinghamshire, UK); D101 macroporous adsorption resin (Tianjin Haiguang Chemical Company, Tianjin, China); ODS (ODS-A-HG 5–50 μm, YMC Co., Kyoto, Japan) TLC: silica gel GF254 (SiO_2_; 400–500 mesh, Qingdao Marine Chemical Factory, Qingdao, China). 2,2-di-phenyl-1-picrylhydrazyl (DPPH) (Sigma Corporation, Ronkonkoma, New York, NY, USA). 2,2′-azino-bis(3-ethylbenzothiazoline-6-sulfonic acid) (ABTS) (Shanghai Sangon Biological Coporation, Shanghai, China). K_2_S_2_O_8_ (Tianjin Guangfukeji Development Corporation, Tianjin, China).

### 3.2. Plant Materials

The roots of *Rhodiola crenulata* (HK. f. *et*.Thoms) H. Ohba were obtained from Shijiazhuang Yiling Pharmaceutical Co., Ltd. (Shijiazhuang, China), and were identified by Dr. Chun-Hua Wang of Tianjin University of Traditional Chinese Medicine. A voucher specimen (No. 20121011CH) was deposited in Tianjin Key Laboratory of Modern Chinese Medicine, Taida, Tianjin, China.

### 3.3. Extraction and Isolation

The roots of *R. crenulata* (25.0 kg) were extracted three times with 95% EtOH (50 L) with heating for 2 h. The solvent was concentrated to give a crude extract (2500 g), which was subjected to column chromatography (CC) (D101 macroporous adsorption resin, EtOH 95%, 50% and 30%). Then 95% EtOH extract (400 g) was subjected to silica gel CC (120 cm × 15 cm) eluted with a gradient of CH_2_Cl_2_/MeOH (100:0–100:20) to afford eight fractions (F1–F8). F1 (5 g) was further separated by silica gel CC with petroleum ether/ethyl acetate (100:0–100:40) to afford eight subfractions F1–1 to F1–8. Subfraction F1–7 (270 mg) was purified by preparative HPLC with MeOH/H_2_O (72:28) to afford compound **1** (42 mg). F2 (42 g) was subjected to silica gel column chromatography eluting with CH_2_Cl_2_/MeOH (100:0–100:10) to afford 42 subfractions. Compound **4** (330 mg) was obtained from F2–2 and compound **5** (120 mg) from F2–10, respectively. Subfractions F2–12 to F2–20 were merged and purified by preparative HPLC with MeOH/H_2_O (58:42) to afford compounds **14** (25 mg) and **15** (33 mg). Subfraction F2–22 (310 mg) was subjected to Sephadex LH–20 column chromatography with CH_2_Cl_2_/MeOH (50:50) and preparative HPLC with MeOH/H_2_O (55:45) to afford compound **12** (44 mg). Subfractions F2–23 to F2–30 were merged and subjected to Sephadex LH–20 column chromatography with CH_2_Cl_2_/MeOH (50:50) and preparative HPLC with MeOH/H_2_O (48:52) to afford compound **13** (24 mg). F2–35 to F2–42 were merged and separated by preparative HPLC with MeOH/H_2_O (44:56) to afford compound **11** (37 mg). F3 (36 g) was separated by silica gel CC with CH_2_Cl_2_/MeOH (100:0–100:20) to afford 34 subfractions. Compound **3** (47 mg) was obtained from F3–1 using a Sephadex LH–20 column with CH_2_Cl_2_/MeOH (50:50). F3–8 (85 mg) was washed with methanol to obtain feathery crystals of compound **16** (33 mg). Fractions F3–6 to F3–13 were merged and subjected to Sephadex LH–20 column chromatography with CH_2_Cl_2_/MeOH (50:50) to obtain compound **2** (58 mg) and a mixture of compounds **2** and **17** (89 mg), which were purified by preparative HPLC with MeOH/H_2_O (55:45) to yield compound **17** (22 mg). F4 (26 g) was separated by ODS CC with MeOH/H_2_O (20:80, 40:60, 60:40 and 80:20) to afford 10 subfractions. Compound **6** (105 mg) was obtained with a needle crystals from F4–1. F4–4 (3.7 g) was subjected to Sephadex LH–20 CC with CH_2_Cl_2_/MeOH (50:50) to give compound **7** (69 mg). F5 (26 g) was subjected to ODS CC with MeOH/H_2_O (20:80, 40:60, 60:40, 80:20) to get 11 subfractions. F5–2 (1.4 g) was separated by Sephadex LH–20 CC with MeOH to afford compound **9** (52 mg) and preparative HPLC with MeOH/H_2_O (33:67) to afford compound **10** (24 mg). F5–4 (1.1 g) was separated by Sephadex LH–20 CC with MeOH to obtain needle crystals to give compound **8** (420 mg).

### 3.4. Compound Characterization

*Rhodiolate* (**1**): Light yellow solid; IR (KBr) ν_max_: 3411, 2925, 2854, 1734, 1708, 1633, 1592, 1514, 1208 cm^−l^; UV (MeOH) λ_max_: 324, 197.5 nm; HRESIMS *m*/*z* 321.1338 [M − H]^−^ (calcd for C_17_H_21_O_6_: 321.1338), ^1^H- (400 MHz) and ^13^C-NMR (100 MHz) data, see Table 1.

*Herbacetin 7-methyl ether* (**2**): Yellow needles; IR (KBr) ν_max_: 3418, 3309, 2922, 1662, 1252 cm^−l^; UV (MeOH) λ_max_: 199.5, 274.5, 331, 380 nm; HRESIMS *m*/*z* 317.0667 [M + H]^+^ (calcd for C_16_H_13_O_7_: 317.0661), ^1^H- (400 MHz) and ^13^C-NMR (100 MHz) data, see Table 1.

*(+)-Syzygiresinol A* (**11**): brown viscous substance; ${\left[\alpha \right]}_{\text{D}}^{25}$ +4.97 (*c* 0.2, MeOH); IR (KBr) ν_max_: 3402, 3280, 2921, 1755, 1650, 1232 cm^−l^; UV (MeOH) λ_max_: 203, 282.5 nm; HRESIMS *m*/*z* 381.0953 [M + Na]^+^ (calcd for C_19_H_18_O_7_ Na: 381.0950), ^1^H- (400 MHz) and ^13^C-NMR (100 MHz) data, see Table 1.

*(7R,8R)-3-Methoxy-8′-carboxy-7′-en-3′,8-epoxy-7,4′-oxyneolignan-4,9-diol* (**12**): White amorphous powder; ${\left[\alpha \right]}_{\text{D}}^{25}$ −4.67 (*c* 0.2, MeOH); IR (KBr) ν_max_: 3438, 2978, 1688, 1612, 1505, 1265, 1125, 1048 cm^−^^1^; UV (MeOH) λ_max_: 202, 222, 288.5, 319 nm; HRESIMS *m*/*z* 381.0950 [M + Na]^+^ (calcd for C_19_H_18_O_7_Na: 381.0950), ^1^H- (400 MHz) and ^13^C-NMR (100 MHz) data, see Table 1.

*(7R,8R)-3-Methoxy-8′-carboxy-7′-en-3′,7-epoxy-8,4′-oxyneolignan-4,9-diol* (**13**): Yellow viscous powder; ${\left[\alpha \right]}_{\text{D}}^{25}$ −9.00 (*c* 0.2, MeOH); IR (KBr) ν_max_: 3427, 2956, 1537, 1242, 1098, 1024 cm^−1^; UV (MeOH) λ_max_: 202, 224, 287.5, 316 nm; HRESIMS *m*/*z* 381.0952 [M + Na]^+^, calcd for C_19_H_18_O_7_Na: 381.0950), ^1^H- (400 MHz) and ^13^C-NMR (100 MHz) data, see Table 1.

### 3.5. Antioxidant Assay

The DPPH method was widely used to evaluate the antioxidant activity [33,34]. We divided them into three groups, including sample group, control group and blank group. In a 96-well microplate, 150 μL of DPPH solution (250 μM) was added to 50 μL of the test sample in methanol at different concentrations. The OD values of the reaction mixtures was recorded at 517 nm using a Flexstation 3 for 30 min at 30 °C. The DPPH-scavenging activity was calculated by the following formula: % scavenging activity = 100 × 1 − (OD_sample_ − OD_blank_)/OD_control_, OD_sample_ = absorbance of sample and DPPH, OD_blank_ = absorbance of sample and methanol, OD_control_ = absorbance of DPPH and methanol. IC_50_, the concentration of sample needed to scavenge 50% of DPPH radical and was obtained by plotting the DPPH-scavenging percentage of each sample against the sample concentration. Ascorbic acid was used as the positive control in this experiment.

The ABTS assay was adopted to evaluate the antioxidant activity of phenolic compounds as well as the free radical-scavenging assay [35]. This assay was based on the oxidation of radical cation ABTS^+^, which was read at 734 nm. The working ABTS reagent was prepared by mixing the same volume of ABTS methanol solution (7 mM) and K_2_S_2_O_8_ solution (6.63 mg to 10 mL 50% methanol). Then diluted with methanol 1:3. The ABTS scavenging activity share the same method. Except that the OD values of the reaction mixtures were recorded at 734 nm using a Flexstation 3 for 10 min at 30 °C.

### 3.6. Cell Culture and Cytokine Assay

The spleen lymphocyte cells obtained from Balb-c mouse were maintained in RPMI 1640 medium containing 10% heat inactivated fetal bovine serum, 1% penicillin-streptomycin at 37 °C in a humidified atmosphere containing 5% CO_2_ and 95% air. For compounds treatment tests, cells were cultured in triplicate in Coster flat-bottom cell culture plates (Corning Inc., Corning, NY, USA). Cells were plated at a density of 2 × 10^6^ cells/well in 24-well cell culture plates. Compounds to be tested were initially dissolved in 10 μL of DMSO, and then RPMI 1640 was added to make solutions in a series of concentration. The final concentration of test compounds was 10 μM. Cells were supplemented with the test compounds as stimulation with 1 μg/mL Con A. The activated cells were further incubated for 48 h. Control cells were grown under indentical conditions but were only exposed to Con A.

After an incubation period (48 h), supernatants of cells were analyzed for IFN-γ secretion. Concentrations of IFN-γ were determined by ELISA kits (eBioscience, San Diego, CA, USA), following the manufacturer instructions.

### 3.7. Cell Viability Test

Cell viability was determined using a CCK-8 cell proliferation and cytotoxicity assay kit (Beyotime Institute of Biotechnology, Haimen, China), according to the supplier’s manual. Dispense 100 μL of cell suspension (5000 cells/well) in a 96-well plate. Pre-incubate the plate for 24 h in a humidified incubator (at 37 °C, 5% CO_2_). Add 10 μL of 100 μM concentrations of compounds to be tested to the plate for 48 h in the incubator. Briefly, at the end of the drug treatment period, 10 μL CCK-8 solutions were fed to each well of the culture plate (containing 100 μL medium). After 4 h incubation, the optical density of the assay solution was measured at 450 nm with a spectrophotometer (BioTek, Winooski, Vermont, VT, USA).

## 4. Conclusions

In summary, the functional food, *R. crenulata* (HK. f. *et*.Thoms) H. Ohba, with its unique medicinal effects, has attracted increasing attention in the food and pharmaceutical fields. In our work, isolation and characterization resulted in two new and 15 known phenolic compounds, which structures were elucidated by chemical and spectroscopic analyses, and the absolute configurations of two lignan isomers were confirmed for the first time by CD analyses. In addition, the ^13^C-NMR data of herbacetin 7-methyl ether was corrected by our group. The biological assay disclosed flavonoids exhibited potent antioxidant activity, but other phenylpropanoids, including the new compounds, showed weak antioxidant activity. Based on the antioxidant evaluation results of the isolated phenolic compounds, we think the various flavonoids are the major chemical constituents responsible for the anti-aging, life-span increasing and anti-radiation activities. To further evaluate the biological activity of these isolated phenols, the induction of IFN-γ production was also evaluated because of its known immunoregulatory activity. As a result, four lignans (compounds **12**, **13**, **14** and **15**) had the ability to induce IFN-γ production as well as two flavonoid glycosides (compounds **8** and **9**), moreover, the new compound **1** also possessed the inducing IFN-γ production ability based on the cytokine assay. In all, the result of phytochemical investigation further reveals the chemical composition of *R. crenulata* and the biological evaluations of these compounds can provide insights into the various bioactivities of the chemical constituents of *R. crenulata*. As to the immunomodulatory effect of *R. crenulata*, polysaccharides of *R. crenulata* will be further investigated by our group to reveal the chemical basis of this activity.

## Supplementary Materials

IR, HR-ESI-MS, ^1^H-NMR, ^13^C-NMR, DEPT-135, ^1^H-^1^H COSY, HSQC, and HMBC spectra for compounds **1**, **2**, **11**–**13** can be accessed at: http://www.mdpi.com/1420-3049/20/08/13725/s1.
