# Supplementary material for: Phenolic Compounds from the Roots of Rhodiola crenulata and Their Antioxidant and Inducing IFN-γ Production Activities
Source: Molecules. 2015 Jul 28;20(8):13725–39. doi: 10.3390/molecules200813725 (PMC6332023; doi:10.3390/molecules200813725)
Supplement: Supplementary file 1 [file molecules-20-13725-s001.pdf]

Supporting Information

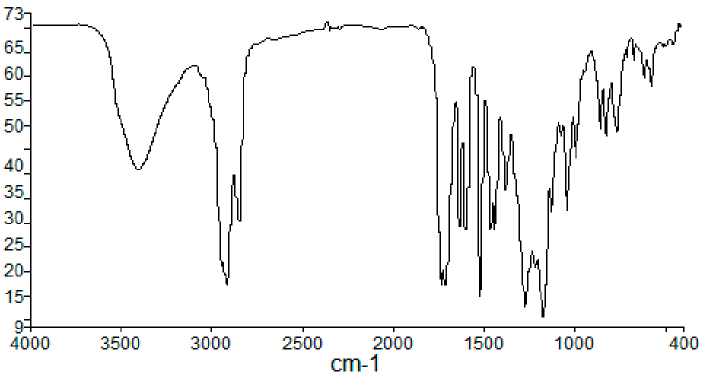

Figure S1. IR spectrum of compound **1**.

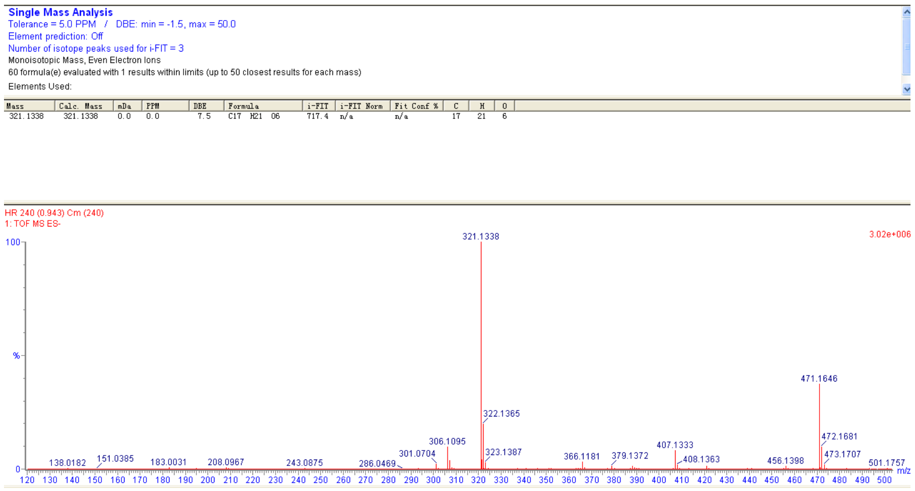

Figure S2. HR-ESI-MS of compound **1**.

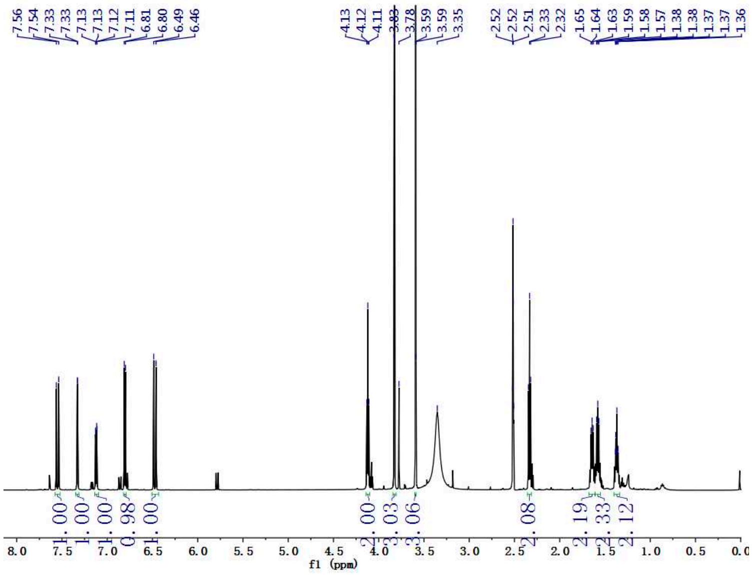

Figure S3. ^{1}H-NMR spectrum of compound **1**.

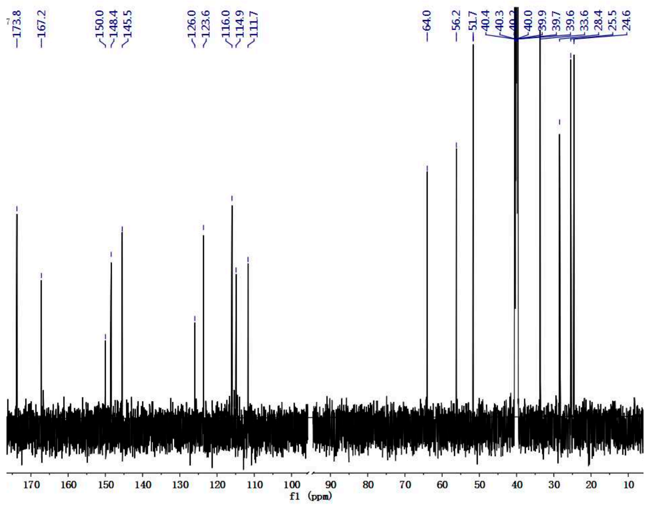

Figure S4. ^{13}C-NMR spectrum of compound **1**.

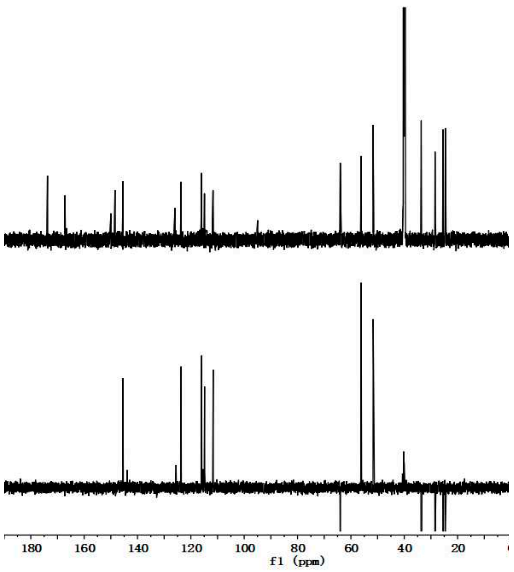

Figure S5. DEPT-135 spectrum of compound **1**.

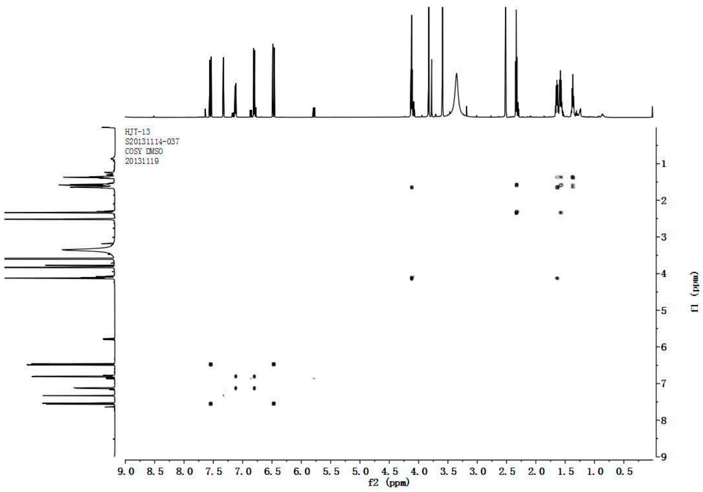

Figure S6. ^{1}H-^{1}H COSY spectrum of compound **1**.

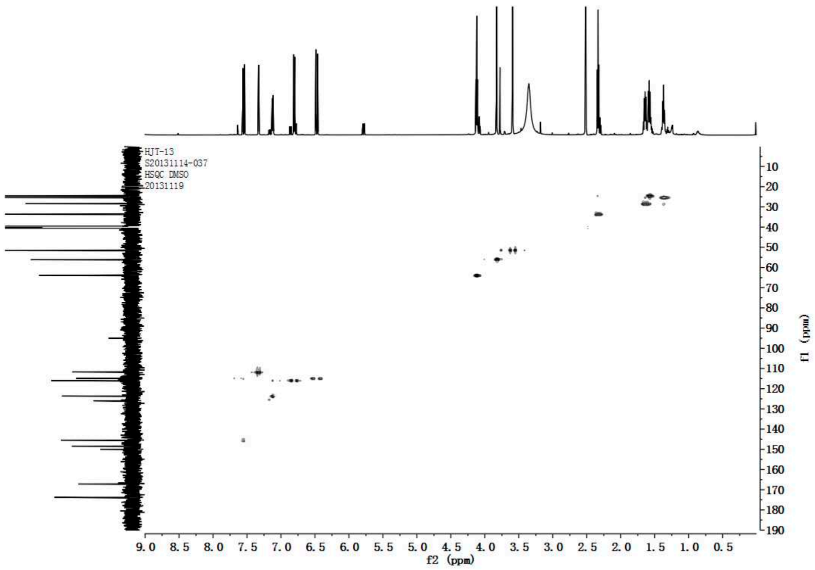

Figure S7. HSQC spectrum of compound **1**.

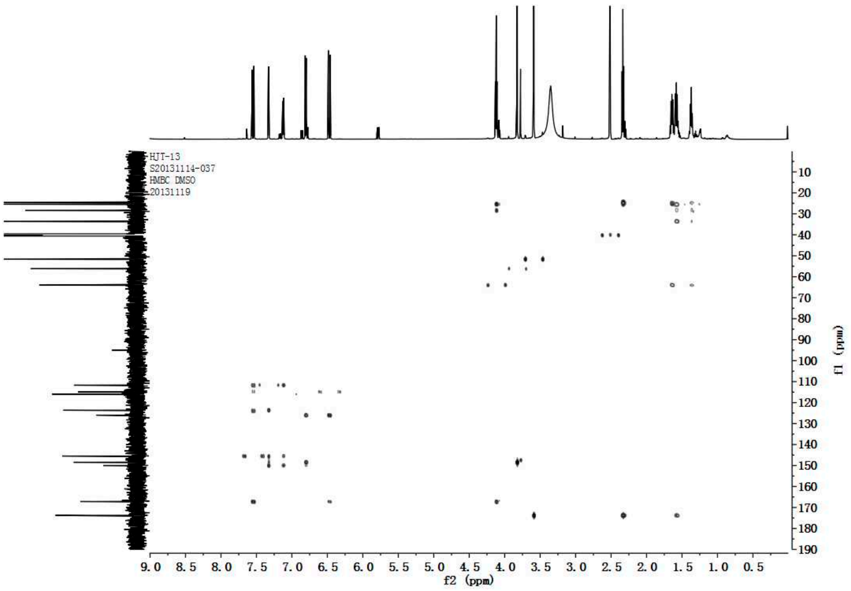

Figure S8. HMBC spectrum of compound **1**.

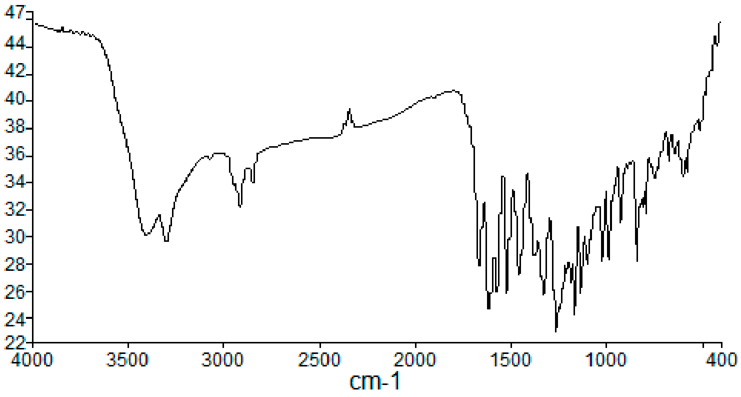

Figure S9. IR spectrum of compound **2**.

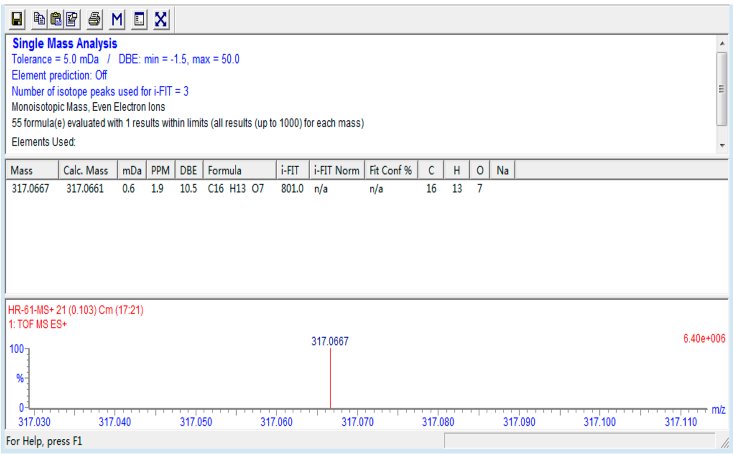

Figure S10. HR-ESI-MS of compound **2**.

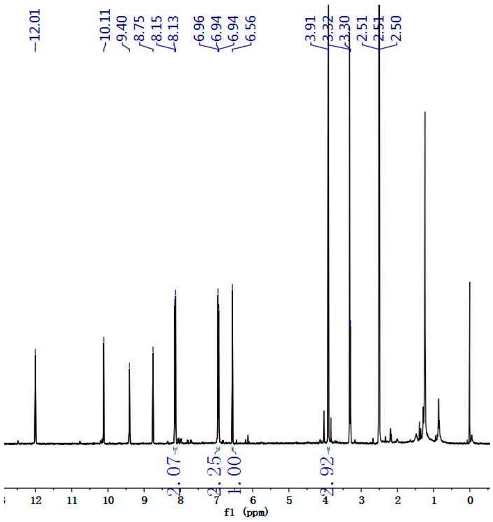

Figure S11. ^{1}H-NMR spectrum of compound **2**.

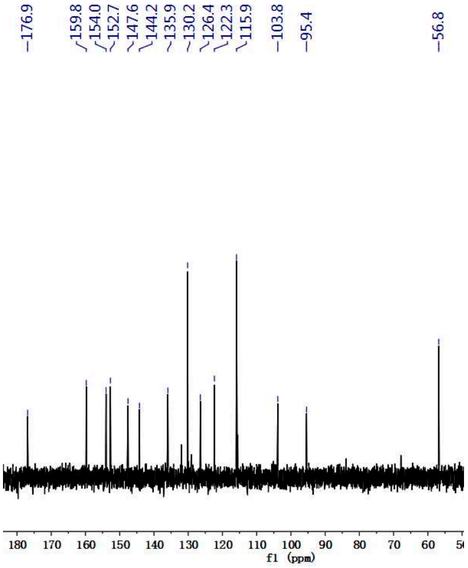

Figure S12. ^{13}C-NMR spectrum of compound **2**.

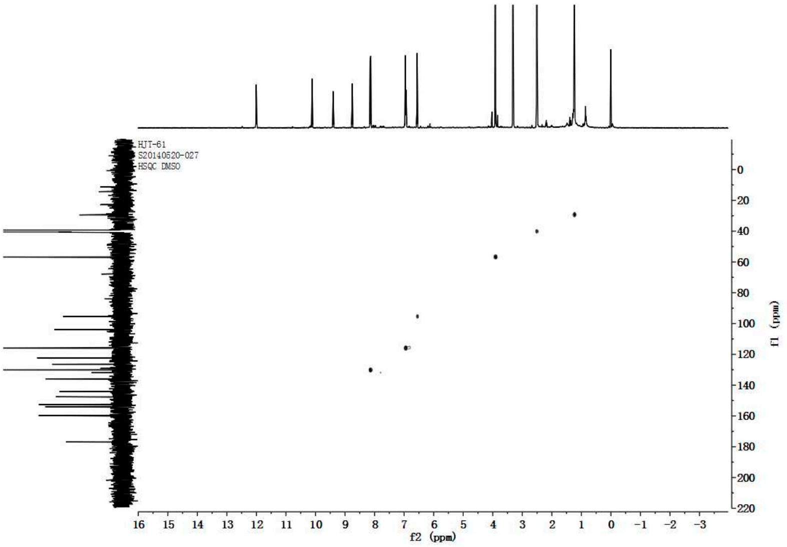

Figure S13. HSQC spectrum of compound **2**.

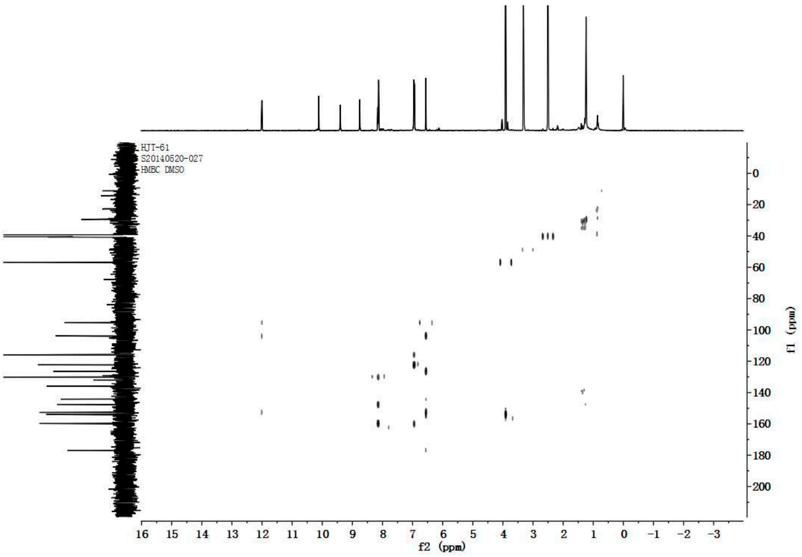

Figure S14. HMBC spectrum of compound **2**.

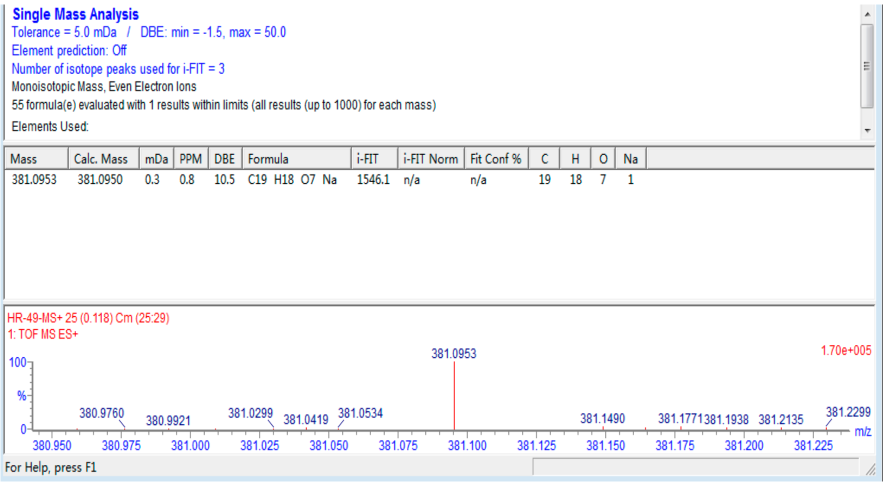

Figure S15. HR-ESI-MS of compound **11**.

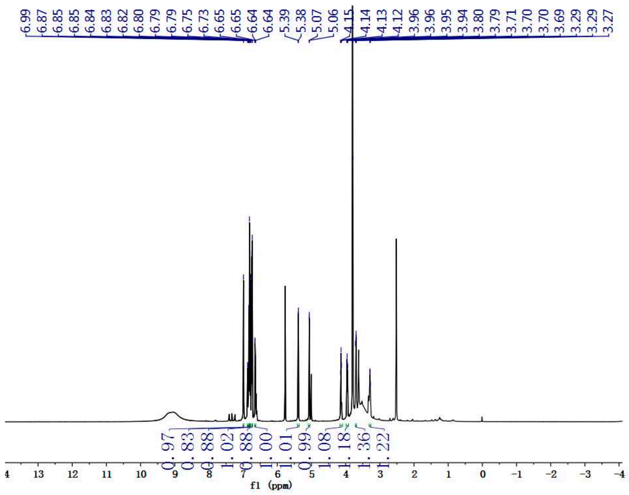

Figure S16. ^{1}H-NMR spectrum of compound **11**.

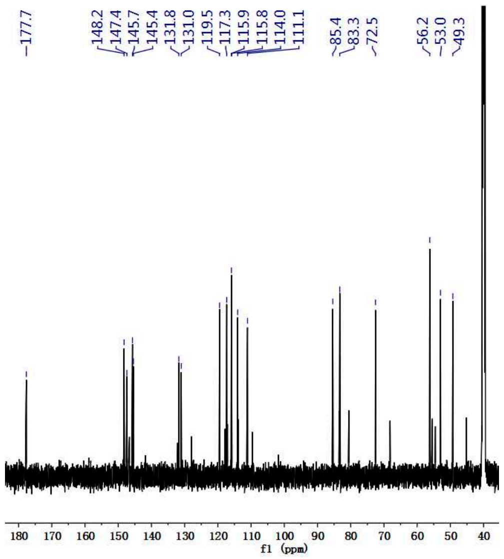

Figure S17. ^{13}C-NMR spectrum of compound **11**.

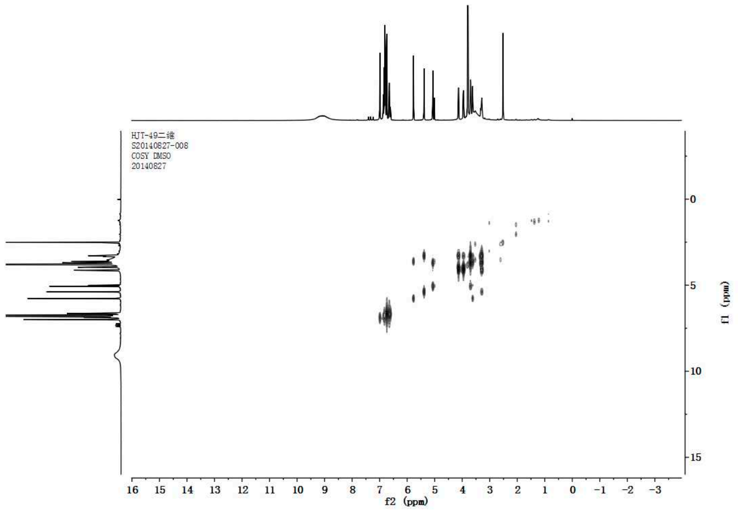

Figure S18. ^{1}H-^{1}H COSY spectrum of compound **11**.

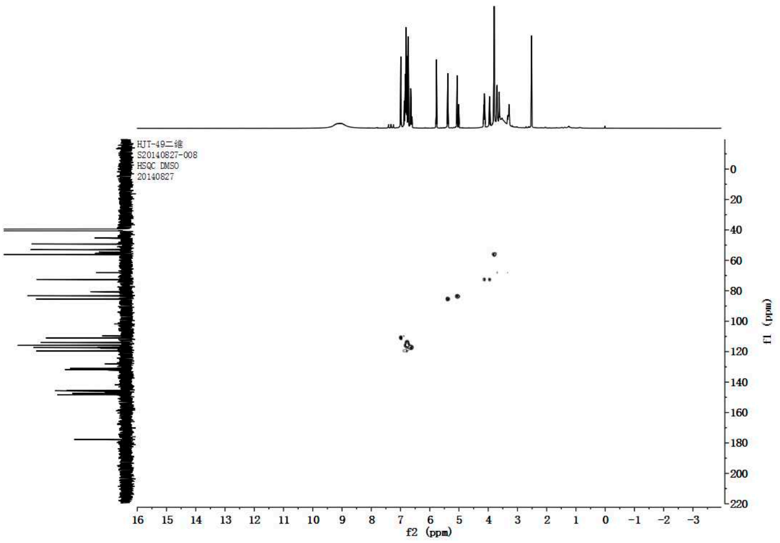

Figure S19. HSQC spectrum of compound **11**.

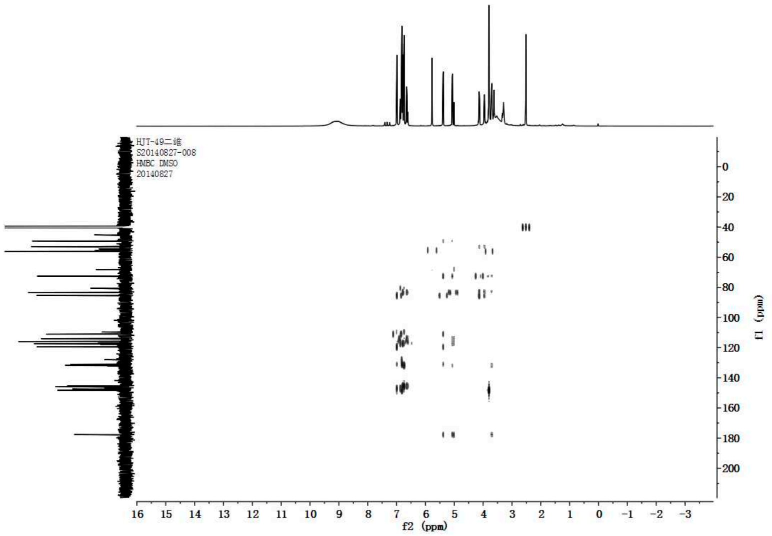

Figure S20. HMBC spectrum of compound **11**.

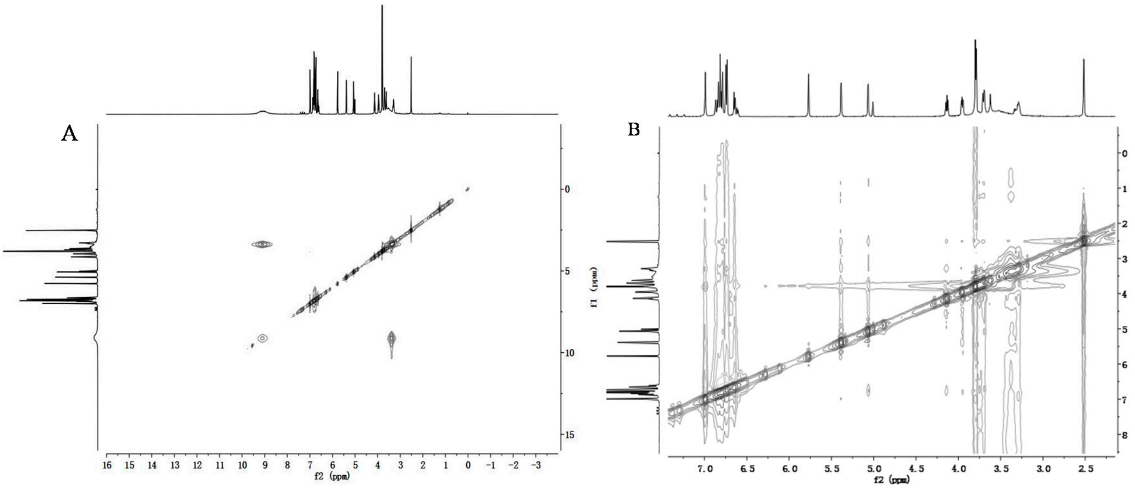

Figure S21. (**A**)(**B**) NOESY spectra of compound **11**.

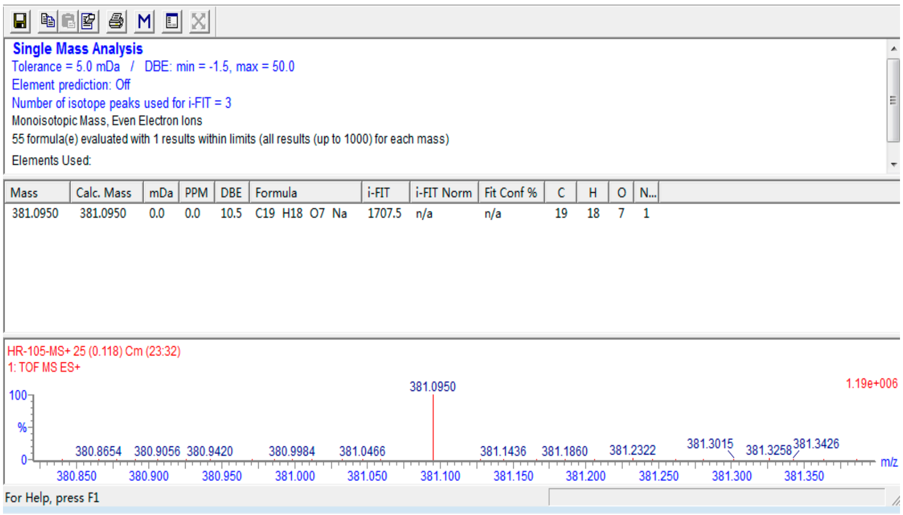

Figure S22. HR-ESI-MS of compound **12**.

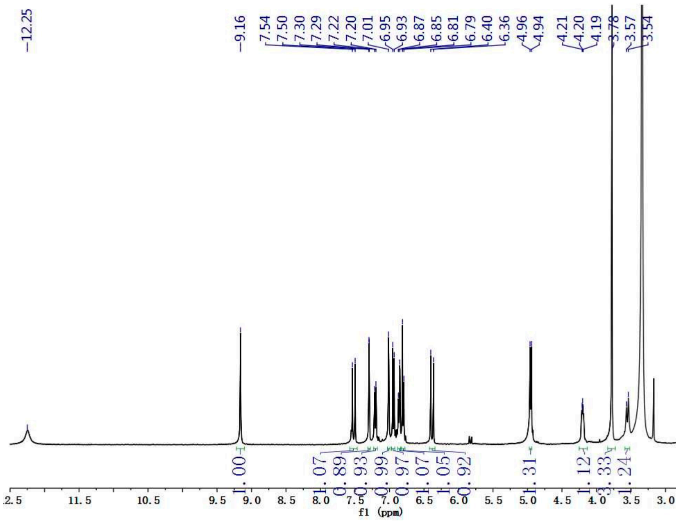

Figure S23. ^{1}H-NMR spectrum of compound **12**.

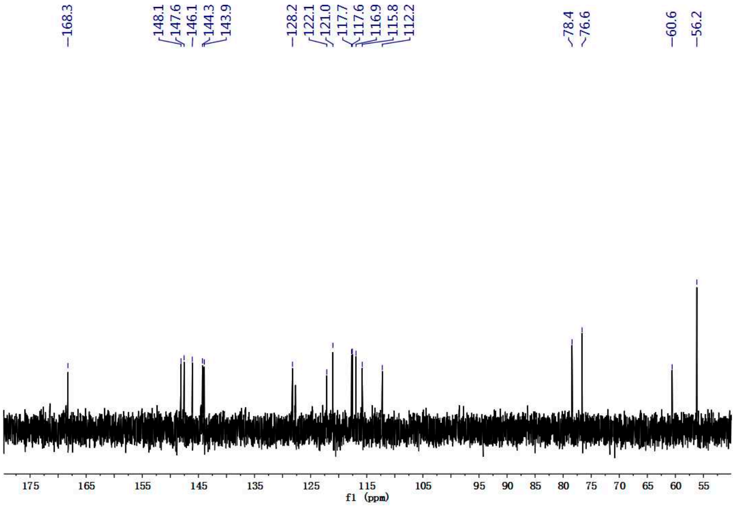

Figure S24. ^{13}C-NMR spectrum of compound **12**.

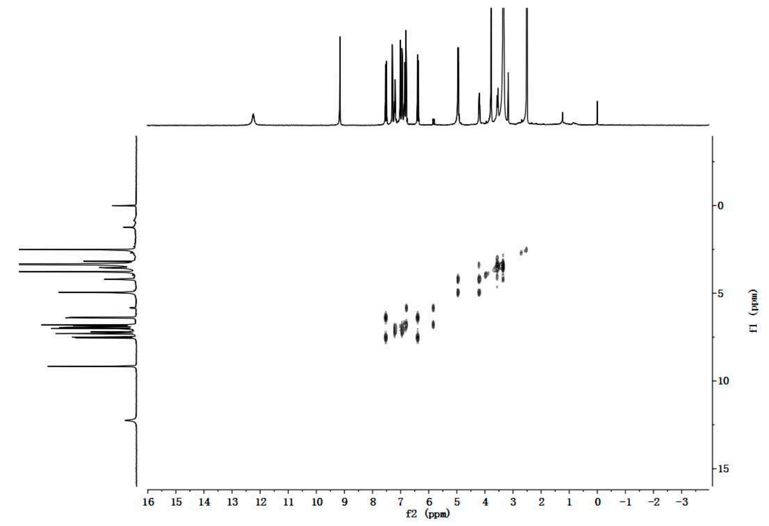

Figure S25. ^{1}H-^{1}H COSY spectrum of compound **12**.

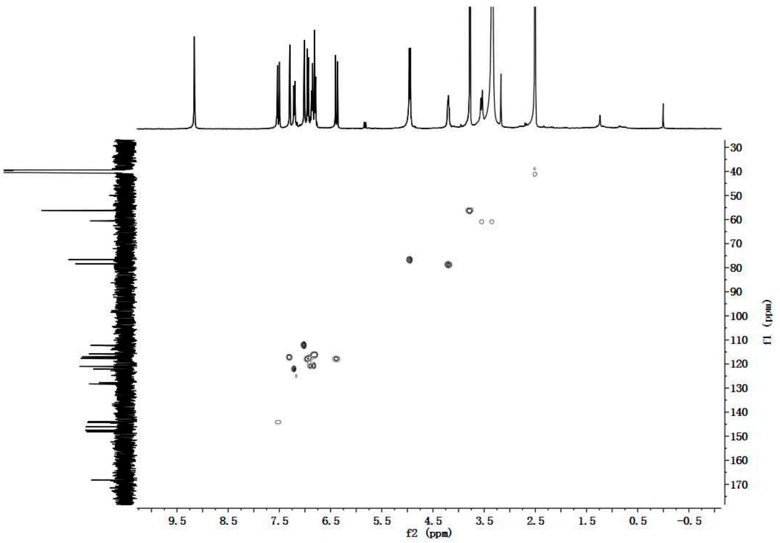

Figure S26. HSQC spectrum of compound **12**.

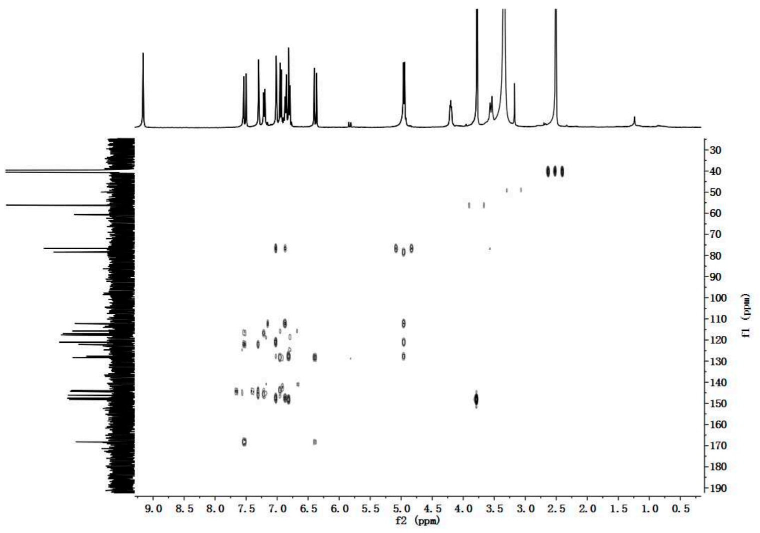

Figure S27. HMBC spectrum of compound **12**.

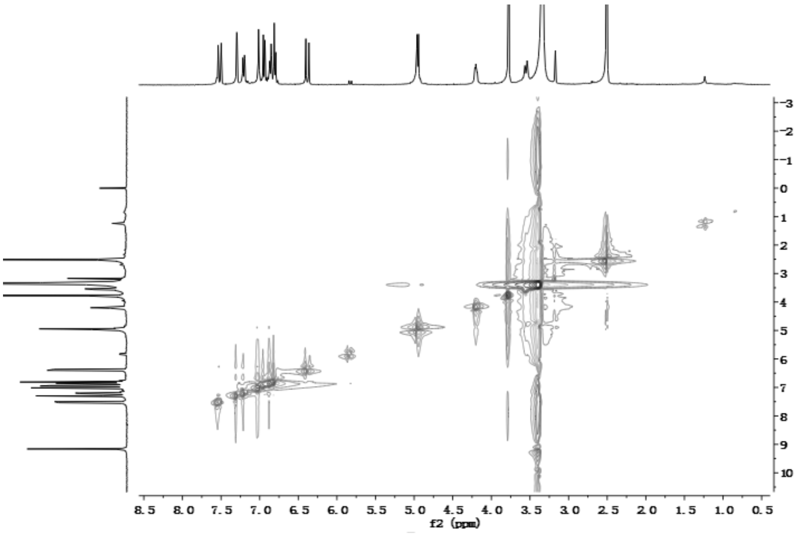

Figure S28. NOESY spectrum of compound **12**.

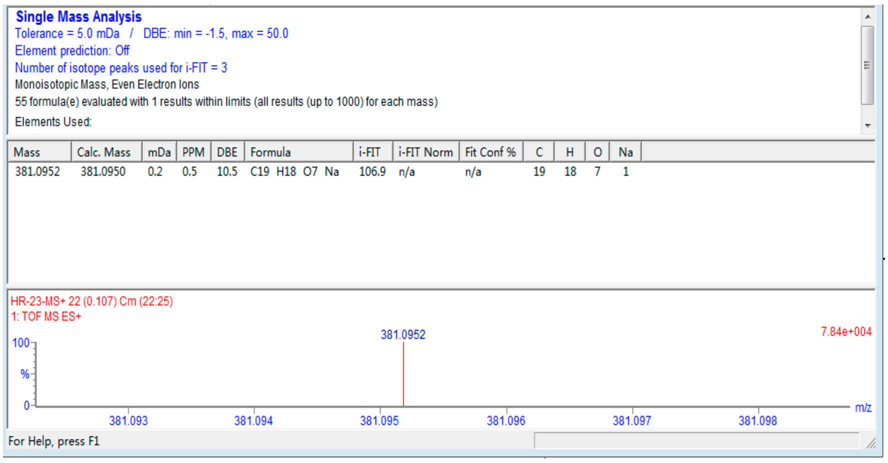

Single Mass Analysis
Tolerance = 5.0 mDa / DBE: min = -1.5, max = 50.0
Element prediction: Off
Number of isotope peaks used for i-FIT = 3
Monoisotopic Mass, Even Electron Ions
55 formula(e) evaluated with 1 results within limits (all results (up to 1000) for each mass)
Elements Used:

Mass	Calc. Mass	mDa	PPM	DBE	Formula	i-FIT	i-FIT Norm	Fit Conf %	C	H	O	Na
381.0952	381.0950	0.2	0.5	10.5	C19 H18 O7 Na	106.9	n/a	n/a	19	18	7	1

HR-23-MS+ 22 (0.107) Cm (22:25)
1: TOF MS ES+
381.0952
7.84e+004
For Help, press F1

Figure S29. HR-ESI-MS of compound **13**.

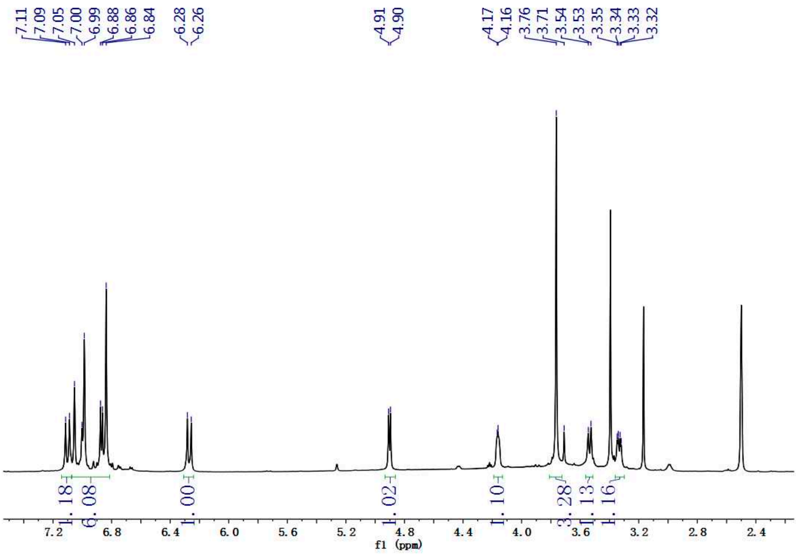

Figure S30. ^{1}H-NMR spectrum of compound **13**.

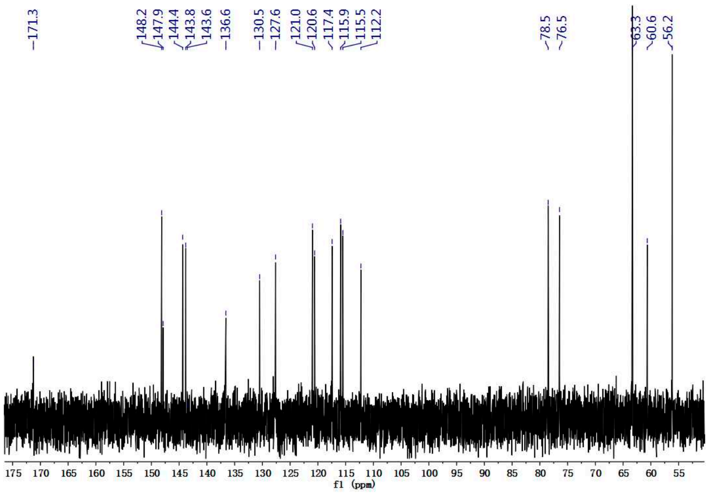

Figure S31. ^{13}C-NMR spectrum of compound **13**.

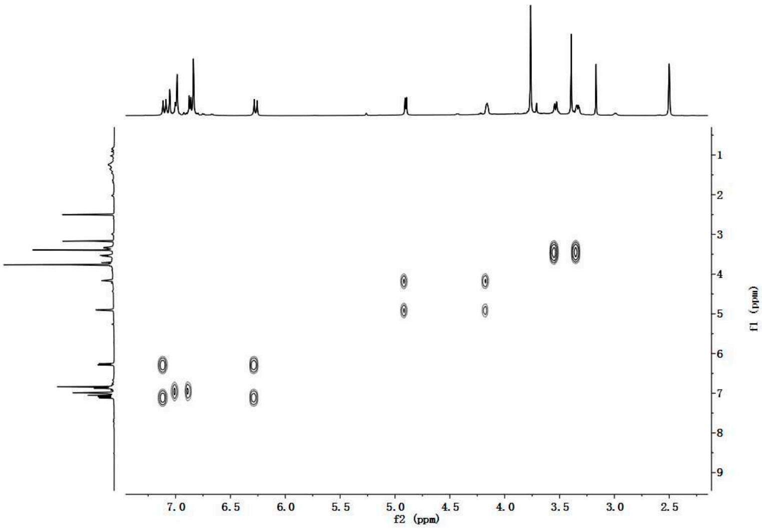

Figure S32. ^{1}H-^{1}H COSY spectrum of compound **13**.

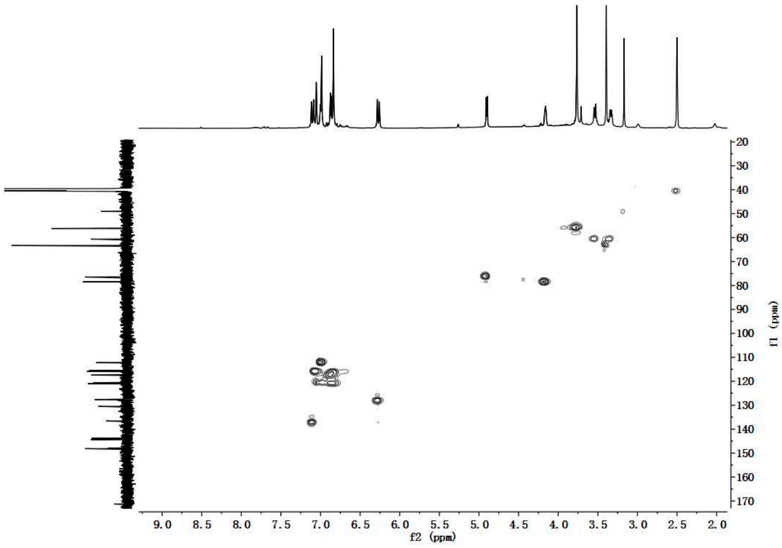

Figure S33. HSQC spectrum of compound **13**.

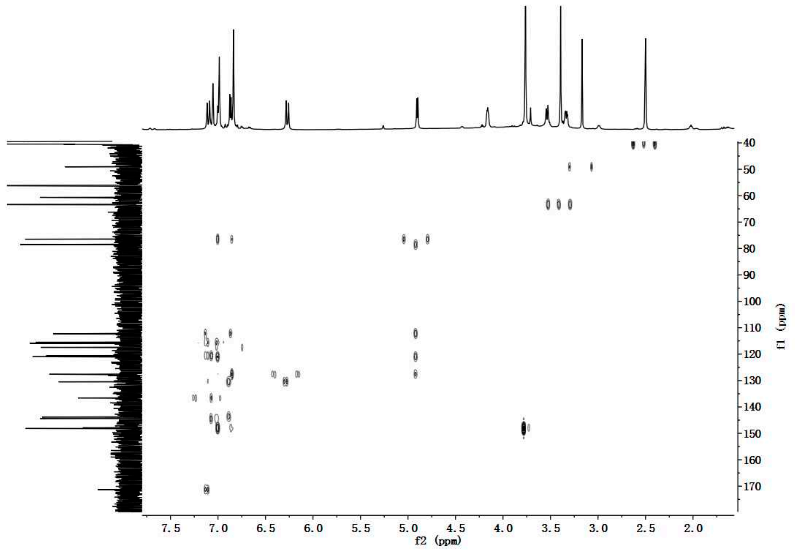

Figure S34. HMBC spectrum of compound **13**.

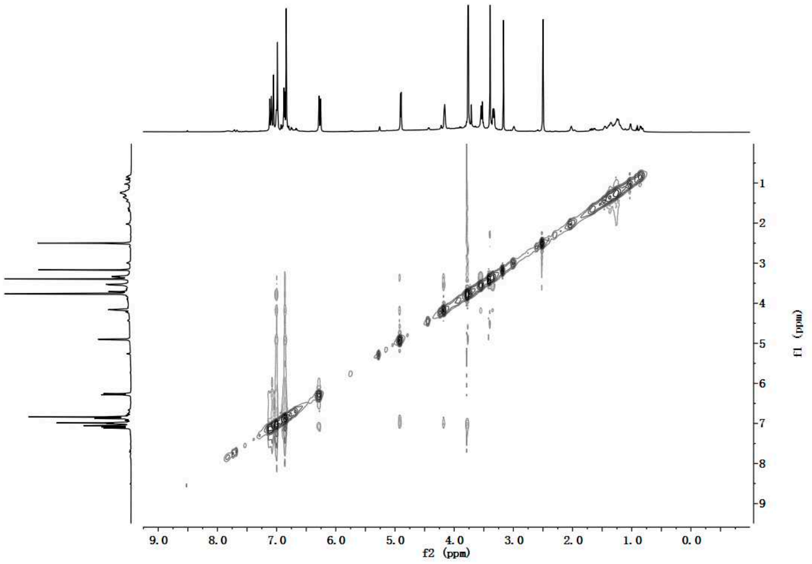

Figure S35. ROESY spectrum of compound **13**.
